# Repeat participation in annual cross-sectional surveys of drug users and its implications for analysis

**DOI:** 10.1186/s13104-018-3454-y

**Published:** 2018-06-04

**Authors:** P. A. Agius, C. K. Aitken, C. Breen, P. M. Dietze

**Affiliations:** 1 Burnet Institute, 85 Commercial Rd, Melbourne, VIC 3004 Australia; 2 School of Public Health and Preventive Medicine, Monash University, 99 Commercial Rd, Melbourne, VIC 3004 Australia; 3 National Drug and Alcohol Research Centre, University of New South Wales, Sydney, NSW 2052 Australia

**Keywords:** Repeat participation, Cross-sectional survey, Point prevalence estimation, Design effect estimation, Population inference

## Abstract

**Objective:**

We sought to establish the extent of repeat participation in a large annual cross-sectional survey of people who inject drugs and assess its implications for analysis.

**Results:**

We used “porn star names” (the name of each participant’s first pet followed by the name of the first street in which they lived) to identify repeat participation in three Australian Illicit Drug Reporting System surveys. Over 2013–2015, 2468 porn star names (96.2%) appeared only once, 88 (3.4%) twice, and nine (0.4%) in all 3 years. We measured design effects, based on the between-cluster variability for selected estimates, of 1.01–1.07 for seven key variables. These values indicate that the complex sample is (e.g.) 7% less efficient in estimating prevalence of heroin use (ever) than a simple random sample, and 1% less efficient in estimating number of heroin overdoses (ever). Porn star names are a useful means of tracking research participants longitudinally while maintaining their anonymity. Repeat participation in the Australian Illicit Drug Reporting System is low (less than 5% per annum), meaning point-prevalence and effect estimation without correction for the lack of independence in observations is unlikely to seriously affect population inference.

## Introduction

Primary health research involving complex sampling often employs inappropriate statistical approaches to inference, and often gives insufficient detail to provide methodological clarity [1, 2]. Related to this is the issue in repeated cross-sectional studies whereby pooled cross-sectional estimation in the presence of repeat responses from the same individuals can yield biased estimates and incorrect estimates of standard error if inappropriate statistical methodology is applied [2, 3]. Typically, failure to account for such lack of independence in observations or clustering will underestimate standard errors, resulting in biased inference which in turn may lead to type I error [4, 5].

Australia’s Illicit Drug Reporting System (IDRS) is an annual survey of people who inject drugs (PWID), designed to provide nationally comparable data about patterns of injecting drug use and related harms and inform future policy and research initiatives [6]. A tacit assumption in the field, and in analysis of IDRS data, has been that similar cohorts of PWID participate in these surveys repeatedly. Given this assumption and the potential problems associated with failure to incorporate a complex sample design into inference estimation, we sought to establish the extent of repeat participation among IDRS participants and assess the implications for reliable analysis of IDRS data.

The work described in this manuscript is the side product of another research project (the IDRS).

## Main Text

### Methods

The IDRS involves a quantitative survey of PWID recruited from capital cities in all Australian states and territories. The same methodology has been employed since 1997 [6]. To be eligible, participants are required to have injected drugs at least monthly in the 6 months preceding interview and to have resided in the same capital city during the previous 12 months. Convenience sampling is facilitated by recruitment notices at needle and syringe programs, staff at these services advising potential participants of the research, and snowballing (i.e., the recruitment of participants’ friends and associates via word of mouth).

The IDRS requires annual ethics approval: the University of New South Wales Ethics Committee approved the national IDRS (# HC12086) in 2015. Informed consent to participate in the study was obtained from all participants.

Although the core of the IDRS questionnaire has varied little since 1997, occasional changes are made to accommodate new issues and facilitate new analyses. Since 2013, IDRS interviewers have been asking participants for their “porn star name” (PSN—the name of their first pet followed by the name of the first street in which they lived; e.g. the second author’s PSN is Sam Banyan) in order to have some ability to assess the overlap in participation across years. PSN has previously been shown to be a unique and reliable identifier [7]. IDRS participants are asked for the information needed to create their PSN as follows: “What was your first pet’s name [if no pet, star sign]?” “What is the name of the first street you ever lived in [or can remember living in]?”

The two names given in each annual IDRS (2013–2015) were exported to separate columns in an Excel file with IDRS years in a third column, sorted alphabetically on first (pet) names, inspected for discrepancies in spelling, punctuation and capitalisation between plausibly matching names, then sorted on second (street) names and inspected again. Names occurring in two or three IDRS iterations were counted and unique IDs assigned to them.

We estimated the effect of repeat observation/participation in terms of the variance associated with parameter estimates by calculating the design effect (DEFF) based on the between-cluster variability for selected prevalence estimates (use of heroin, last 6 months and ever; use of crystal methamphetamine (‘ice’), last 6 months and ever; injected with a needle used by someone else, last month; number of heroin overdoses, ever; number of injections, last month). The DEFF represents the ratio of the variance of the complex estimator (i.e., accounting for participant clustering from repeated observations) to that assuming prevalence was estimated on truly independent observations from a simple random sample (SRS). For comparative purposes and given the convenience sampling used in the IDRS, we also estimated the sample DEFF using jackknife re-sampling variance estimation [8], essentially deriving the ratio of the variance from the jackknife variance estimator, accounting for participant clustering to jackknife variance estimation assuming no repeat observations. (Jackknife variance estimation is a data-dependent estimation method (i.e., not based on normal theory) which estimates variance between point estimates from a process of iterative data re-sampling (based on the number of sample units in the sample), where in each re-sampled set of data one observation (either an individual response or a set of responses in the case of estimation accounting for participant clustering) is omitted.) Univariate sample means (proportions for dichotomous measures) were produced to estimate prevalence for each factor. Taylor-linearised standard errors were used to report 95% confidence intervals about point estimates, taking account of the lack of independence in observations [9]. Analyses were undertaken using SPSS (version 22) and Stata (version 13.1) statistical packages.

### Results

Eight hundred and eighty-six, 898 and 887 IDRS participants supplied PSNs in 2013, 2014 and 2015 respectively, giving 2565 unique names. Across the three IDRS samples, 2468 PSNs (96.2%) appeared only once, 88 (3.4%) twice (“doubles”), and nine (0.4%) in all 3 years (“triples”), giving a mean of 1.04 responses per participant. Of the 88 doubles, 79 (89.8%) occurred in consecutive years. Including triples, 29 names (1.1%) appeared in both 2013 and 2015. Forty-four PSNs in the 2014 IDRS (4.9%) were observed in 2013, and 43 names in the 2015 IDRS (4.8%) in 2014. The low incidence of repeat observations across three successive sets of IDRS participants suggests the sample is almost entirely renewed every 2 years. Table 1 shows the estimated prevalences of selected behaviours across 2013–15 and their accuracy.

**Table 1:** Prevalences of selected behaviours, 2013–2015: mean, 95% confidence interval (95% CI) and design effect (DEFF)

Self-reported behaviour	Mean^a^	95% CI	DEFF1^b^	DEFF2^c^
Used heroin, ever (n = 2565)	.88	(.86, .89)	1.07	1.07
Used heroin, last 6 months (n = 2565)	.59	(.57, .61)	1.06	1.05
Used ice, ever (n = 2562)	.81	(.79, .82)	1.06	1.06
Used ice, last 6 months (n = 2564)	.61	(.59, .63)	1.04	1.04
Injected with a needle used by someone else, last month (n = 2489)	.07	(.06, .08)	1.02	1.02
No. of heroin overdoses, ever (n = 2081)	2.3	(1.8, 2.7)	1.01	1.01
No. of injections, last month (n = 2426)	38.3	(36.1, 40.5)	1.03	1.03

^a^ Indicates proportions for binary measures and average counts for interval measures

^b^ DEFF1 = ratio of the variance of the complex estimator to that from estimation assuming SRS (e.g. DEFF of 1.06 indicates the complex sample is 6% less efficient in estimating prevalence than an SRS)

^c^ DEFF2 = ratio of the variance of the jackknife estimator accounting for participant clustering in response to that from jackknife estimation without accounting for clustering

### Discussion

The finding of negligible overlap between IDRS samples lends support to the notion that Australian PWID ageing is a population effect rather than a sample-specific one [6], and means that point-prevalence and effect estimation without correction for the lack of independence in observations is unlikely to seriously affect population inference. Nonetheless, as our analysis shows, repeated cross-sectional IDRS samples do exhibit a small degree of repeat observation across periods and this does inflate standard error marginally when estimating prevalence. This research also demonstrates that using a participant-generated anonymous unique identifier is an effective means by which to identify participant clustering in repeated cross-sectional data and can be used to estimate the degree of non-independence in sampling and correct standard errors if necessary. Despite the evidence that the level of non-independence of samples is low, in light of this lack of independence, appropriate and more conservative methods of estimation of standard error (e.g. Taylor-linearised [9], cluster robust [10] jackknife [8] or bootstrapped [11] standard errors) should be used where possible. Furthermore, where more complex variance estimators are used in the estimation of standard error, it is important that the methodological approach be detailed comprehensively in published work in order to inform assessment of the quality of the research and to provide guidance for those with similar data [12].

## Limitations

More than 10% of IDRS participants in each year did not supply a PSN, affecting the accuracy of our estimates to an unknown extent. Several participants reported no first pet so gave a star sign instead (resulting in PSNs such as “Cancer Unknown”), and several unusual street names (from the same city) were repeated but accompanied by a pet name in 1 year and a star sign in another, which we regarded as denoting different individuals. It is possible that these data mean we have underestimated repeat participation, but their rarity means they have only a slight effect. Conversely, some of the few combinations we assessed as identical (e.g. Satan Holmes/Homes) might have been from separate individuals, thus overestimating repetition. Careful programming, such as probability-based matching methods/algorithms (e.g. fuzzy matching [13], soundex code [14]), would be needed to match misspelt names in larger datasets efficiently and to quantify the degree of error that is associated with matching.

One should also note that the IDRS is a non-probability sample, and in comparing standard errors for complex and SRS estimators, variance estimates from the SRS estimator assume random sampling from a specific population frame. However, given there was virtually no difference in DEFF estimates using data-dependent jackknife re-sampling estimation, we expect that this will have negligible effect. Furthermore, readers should note that the analyses undertaken in this research are strictly exploratory and secondary to the aims of IDRS data collection and reporting.

## Abbreviations

CI: confidence interval
DEFF: design effect
IDRS: Illicit Drug Reporting System
PSN: porn star name
PWID: people who inject drugs
SRS: simple random sample
